# Compositional Effects on the Tensile Behavior of Atomic Bonds in Multicomponent Cu_93−x_Zr_x_Al_7_ (at.%) Metallic Glasses

**DOI:** 10.3390/molecules30122602

**Published:** 2025-06-16

**Authors:** Tittaya Thaiyanurak, Olivia Gordon, Muyang Ye, Zhengming Wang, Donghua Xu

**Affiliations:** 1 Materials Science Program, Oregon State University, Corvallis, OR 97331, USA; 2 School of Mechanical, Industrial and Manufacturing Engineering, Oregon State University, Corvallis, OR 97331, USA; 3 School of Arts and Sciences, Atlanta Metropolitan State College, Atlanta, GA 30310, USA; 4 Department of Computer Science, University of Southern California, Los Angeles, CA 90007, USA

**Keywords:** atomic bonds, metallic glass, compositional effect, tensile behavior, multicomponent, mechanical properties, alloys, amorphous materials

## Abstract

The mechanical properties of materials are fundamentally determined by the behavior of atomic bonds under stress. Probing bond behavior during deformation, however, is highly challenging, particularly for materials with complex chemical compositions and/or atomic structures, such as metallic glasses (MGs). As a result, a significant gap exists in the current understanding of the mechanical properties of MGs in relation to the atomic bond behavior and how this relationship is influenced by metallurgical factors (e.g., alloy composition, processing conditions). Here, we present our study of the compositional effects on the tensile behavior of atomic bonds in Cu_93−x_Zr_x_Al_7_ (x = 40, 50, 60 at.%) MGs using large-scale molecular dynamics (MD) simulations and statistical analysis. Specifically, we examine the populations (fractions), mean bond lengths, mean bond z-lengths, and mean bond z-strains of the different bond types before and during tensile loading (in the z-direction), and we compare these quantities across the different alloy compositions. Among our key findings, we show that increasing the Zr content in the alloy composition leads to shortened Zr-Zr, Al-Cu, Al-Zr, and Cu-Zr bonds and elongated Cu-Cu bonds, as evidenced by their mean bond lengths. During deformation, the shorter Zr-Zr bonds and longer Cu-Cu bonds in the higher-Zr-content alloys, compared with those in the x = 40 alloy, appear stronger (more elastic stretching in the z-direction) and weaker (less z-stretching), respectively, consistent with general expectations. In contrast, the Al-Cu, Al-Zr, and Cu-Zr bonds in the higher-Zr-content alloys appear weaker in the elastic regime, despite their shortened mean bond lengths. This apparent paradox can be reconciled by considering the fractions of these bonds associated with icosahedral clusters, which are known to be more resistant to deformation than the rest of the glassy structure. We also discuss how the compositional effects on the bond behavior relate to variations in the overall stress–strain behavior of the different alloys.

## 1. Introduction

**Figure 1:**
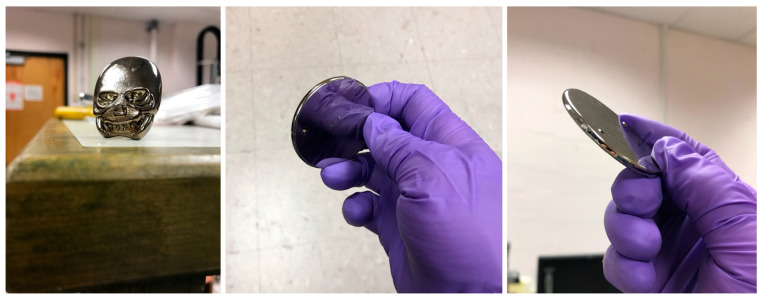
Representative casting products of MGs (with various compositions, different from the Cu-Zr-Al ternary alloys studied in the present work; shown for illustration purposes): (**left**) a skull with a complex shape and many fine details replicated from the mold, including roughness in some areas; (**middle** and **right**) a round disk with a 50 mm diameter and 3 mm thickness, featuring smooth and reflective surfaces.

Materials are composed of numerous atoms bonded together. The behavior of the atomic bonds under applied stress fundamentally determines the material’s mechanical properties. While the stress-driven bond behavior is relatively well understood in materials with simple chemical compositions (e.g., pure elements) and simple atomic structures (e.g., single crystal phases), such understanding is limited in more complex materials, such as metallic glasses (MGs). MGs are a class of advanced alloys characterized by their amorphous atomic structure, lacking the long-range order found in traditional crystalline metals. This unique structure imparts MGs with a combination of desirable properties, such as very high strength and hardness, excellent wear resistance, and superior corrosion resistance, making them strong candidates for applications in aerospace, electronics, and biomedical devices [1,2,3,4,5,6,7,8,9,10,11,12,13,14,15,16,17]. Additionally, the amorphous nature of MGs endows them with excellent near-net-shape castability, owing to the absence of crystallization shrinkage during the liquid-to-glass transition. This property is advantageous for the cost- and energy-efficient manufacturing of commercial products. Figure 1 shows representative casting products of MGs, manufactured in the Materials Modeling and Development Lab at Oregon State University (https://research.engr.oregonstate.edu/mmdg, accessed on 17 May 2025).

On the other hand, the disordered structure of MGs—with randomly oriented atomic bonds—and their typical multicomponent compositions (and thus mixed bond types) make their mechanical behavior more complex and less well understood, particularly in relation to stress-driven atomic bond behavior. Diffraction and scattering experiments on MGs can provide useful information about the overall structure—averaged over all types of atomic bonds—with or without applied stress [18,19,20]. However, it is highly challenging to unambiguously deconvolute such data into details about individual atomic bond types. Recently, we demonstrated a computational method that combines molecular dynamics (MD) simulations and detailed statistical analysis of the bond length, bond z-length, and bond z-angle, where z is the uniaxial loading direction [21]. Using this method, we were able to reveal the non-equal contributions of different atomic bonds to both the pre- and post-yielding strength and deformation in an exemplary MG, Cu_46_Zr_47_Al_7_ (at.%) [21]. In particular, we found that the weak bonds (Al-Al, Cu-Cu) yielded much earlier than the intermediate (Zr-Zr, Al-Cu) and strong (Al-Zr, Cu-Zr) bonds and that the overall material’s yielding behavior and post-yielding residual strength and deformation were primarily governed by the intermediate and strong bonds.

In the present study, we use the newly developed computational method to investigate the effects of the alloy composition on the tensile behavior of the various atomic bonds in Cu_93−x_Zr_x_Al_7_ (x = 40, 50, 60 at.%) MGs (hereafter referred to as Zr40, Zr50, and Zr60, respectively). The alloy composition is one of the metallurgical factors that can be readily employed to control the mechanical properties of materials. Understanding how the alloy composition affects the stress-driven bond behavior is directly relevant to practical alloy design towards better mechanical properties. The selected alloy series covers both Cu-based (Zr40) and Zr-based (Zr50, Zr60) MGs within the same Cu-Zr-Al ternary alloy system.

More specifically, we examine how the compositional variation in this alloy series alters the populations (fractions), mean bond lengths, mean bond z-lengths, and mean bond z-strains of the different bond types before and during tensile loading (in the z-direction). With these quantitative data, we discuss the mechanisms underlying the apparent changes in the elastic stretching, yielding, and post-yielding plastic behavior of the different atomic bonds corresponding to the compositional variation. To supplement the discussion, short-range ordering in the form of icosahedral (ICO) clusters, with dependence on the alloy composition, is also considered. We also discuss the relationship between the composition-dependent bond behavior and the variations in the overall stress–strain behavior of the different alloys.

## 2. Results and Discussion

As described in more detail in our previous publication [21], the radial distribution function (RDF) is a commonly used tool to characterize the atomic structures of crystalline and non-crystalline materials. An RDF is evaluated as the atomic number density within a radial distance of r to r+dr from an average atom, normalized by the overall atomic number density in the system, i.e., $g\left(r\right)=\frac{\rho (r)}{\rho_{ideal}}=\frac{dN(r)}{\rho_{ideal}4\pi r^{2}dr}$, where $\rho_{ideal}=N_{tot}/V_{tot}$, dN(r) is the count of atoms within the radial bin from r to r+dr, $N_{tot}$ is the total number of atoms in the material, and $V_{tot}$ is the sample volume. When this evaluation is performed for a selected type of elemental pair only (e.g., Cu-Cu, Cu-Zr), the result is known as a partial RDF. The valley between the first and second peaks on a partial RDF indicates the maximum extent of the first coordination shell between the two elements, which can be used as the cutoff distance for identifying the specific types of bonds.

**Figure 2:**
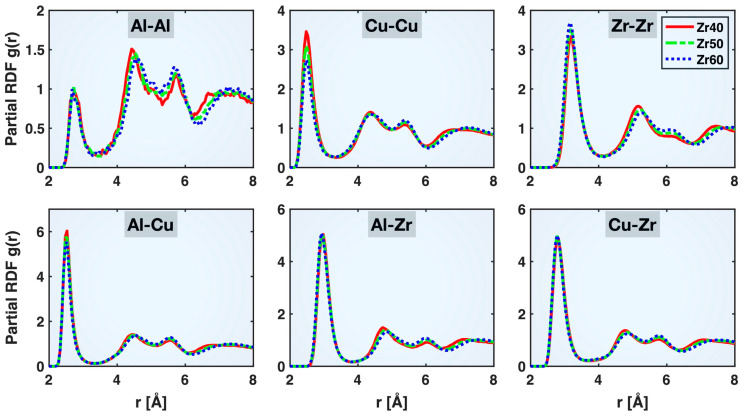
Partial radial distribution functions (RDFs) of different atomic pairs in the relaxed Zr40, Zr50, and Zr60 MGs prior to the tensile test.

Figure 2 compares the partial RDFs of different atomic pairs in the relaxed Zr40, Zr50, and Zr60 MGs prior to the tensile test. Despite some noticeable shifts in the longer-distance portions of the partial RDFs with varying alloy compositions, the valley between the first and second peaks remains nearly unchanged, indicating that a consistent set of cutoff distances can be applied to identify atomic bonds across different compositions. These distances are 3.4, 3.2, 4.1, 3.3, 3.9, and 3.7 Å for Al-Al, Cu-Cu, Zr-Zr, Al-Cu, Al-Zr, and Cu-Zr, respectively. These values are the same as those obtained in the Cu_46_Zr_47_Al_7_ MG previously [21], further indicating that the bonding cutoff distances are rather insensitive to the alloy composition within the Cu-Zr-Al system. We also compared the partial RDFs at the beginning and end of the tensile test and found no appreciable change in the bonding cutoff distances, consistent with observations from our previous study [21]. This suggests that the cutoff distances listed above can be reliably applied to identify the various bonds throughout the tensile deformation process.

**Figure 3:**
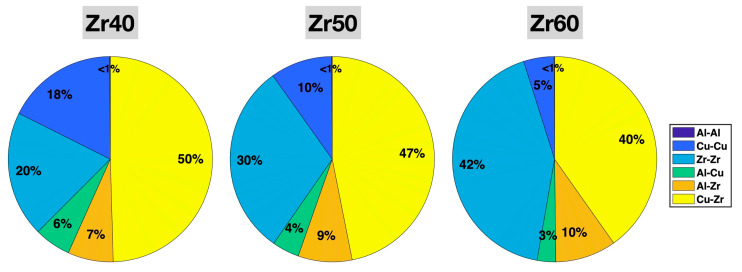
Populations (fractions) of different bonds in the relaxed Zr40, Zr50, and Zr60 MGs prior to the tensile test. The percentages have been rounded to the nearest integer.

Figure 3 shows the populations (fractions among all bonds) of the six different types of bonds in the relaxed Zr40, Zr50, and Zr60 MGs prior to the tensile test. In all three MGs, Zr-Zr and Cu-Zr are the two most abundant bond types, and Al-Al is the least abundant (~0.1%). With increasing Zr content from 40% to 60% in the alloy composition, the Zr-Zr bond fraction increases significantly, from 20% to 42%, while the fractions of all Cu-containing bond types decrease—Cu-Zr from 50% to 40%, Cu-Cu from 18% to 5%, and Al-Cu from 6% to 3%. The Al-Zr bond fraction slightly increases from 7% to 10%. All these changes in the bond fractions are consistent with expectations based on the variations in the alloy composition from Zr40 to Zr60—increasing Zr and decreasing Cu, while fixing the Al content. Given the very small fraction of Al-Al bonds (0.115% in Zr40, 0.106% in Zr50, and 0.097% in Zr60), which may limit their statistical significance, we focus on the other five bond types in the subsequent statistical analysis.

**Figure 4:**
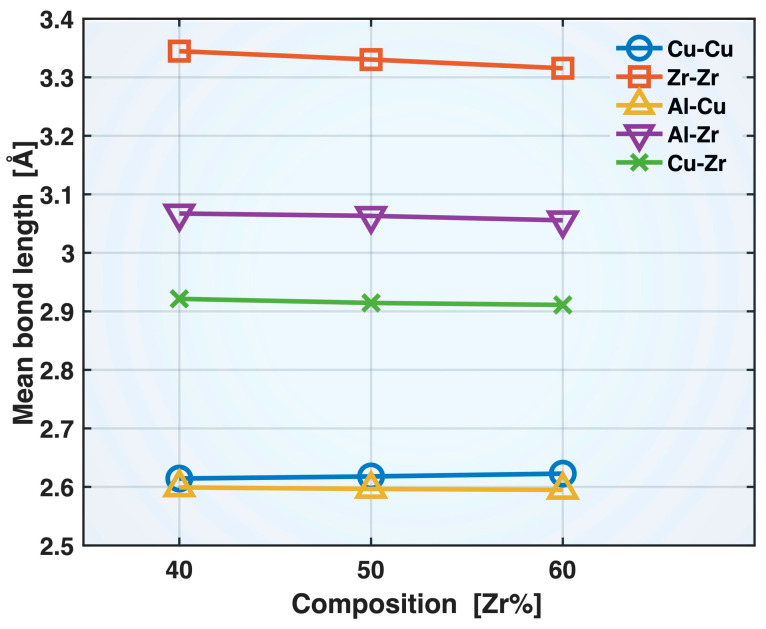
Mean bond lengths of different bonds in the relaxed Zr40, Zr50, and Zr60 MGs prior to the tensile test.

Figure 4 presents the mean bond lengths of the Cu-Cu, Zr-Zr, Al-Cu, Al-Zr, and Cu-Zr bonds in the relaxed Zr40, Zr50, and Zr60 MGs prior to the tensile test. All these bonds exhibit shortening with increasing Zr content in the alloy composition, except for Cu-Cu, which shows moderate elongation. Among the shortened bonds, Zr-Zr exhibits the greatest degree of shortening, with the mean bond length decreasing from 3.345 Å (Zr40) to 3.315 Å (Zr60), while Al-Cu displays the smallest change, from 2.599 to 2.595 Å. The shortening of the Zr-Zr, Al-Cu, Al-Zr, and Cu-Zr bonds can be partly attributed to enhanced cohesion resulting from the increased Zr content in the alloy composition. Zr has high self-cohesion energy (6.25 eV/atom) and large negative heat of mixing with Al (−44 kJ/mol) and Cu (−23 kJ/mol) [22,23]. The increased population, and hence availability, of Zr atoms may have also contributed to the shortening of the Zr-Zr, Al-Zr, and Cu-Zr bonds. On the other hand, the elongation of the Cu-Cu mean bond length may be attributed to the reduced population of Cu atoms and, simultaneously, to Cu’s relatively low self-cohesion energy (3.49 eV/atom).

It is noteworthy that the mean bond lengths presented in Figure 4 for the different bond types all exceed the first peak positions in the corresponding partial RDFs shown in Figure 2. This shows that, although the full first peak of a partial RDF is closely related to the range of bonding distances, its peak position does not represent the mean bond length. This is partly because the RDF is defined differently from a direct histogram of bond lengths and partly because its first peak (as well as some others) has an asymmetric shape, with a more gradual decline on the right side. A more accurate RDF-based evaluation of the mean bond length is $\frac{\int r\;dN(r)}{\int dN(r)}=\frac{\int r\;g\left(r\right)\;\rho_{ideal}4\pi r^{2}dr}{\int g\left(r\right)\rho_{ideal}4\pi r^{2}dr}\approx \frac{\sum g\left(r_{i}\right)\times r_{i}^{3}}{\sum g\left(r_{i}\right)\times r_{i}^{2}}$, where the integrals are approximated using summations over all the RDF data points within the first peak range. This method results in good agreement with the mean bond lengths from direct bond statistics.

Based on the correlations between the mean bond lengths and alloy composition in Figure 4, one might expect that, with increasing Zr content, the Zr-Zr, Al-Cu, Al-Zr, and Cu-Zr bonds would become stronger and more elastic during deformation due to bond shortening, while Cu-Cu would become weaker and less elastic due to bond elongation. As we will show later in this paper, while these expectations hold true for Zr-Zr and Cu-Cu bonds, the composition-induced changes in the deformation behavior of the dissimilar bond types, Al-Cu, Al-Zr, and Cu-Zr, are more complex and cannot be explained solely by bond shortening.

**Figure 5:**
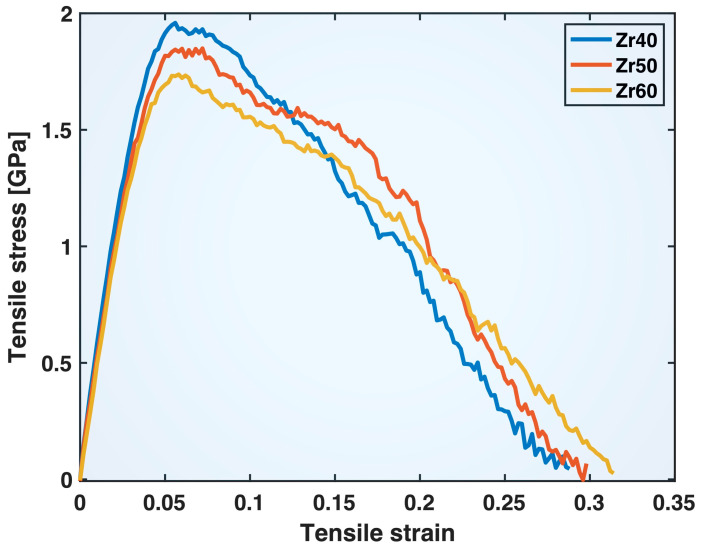
Tensile stress–strain curves of the Zr40, Zr50, and Zr60 MGs in the MD simulations.

Figure 5 shows the tensile stress–strain curves of the Zr40, Zr50, and Zr60 MGs obtained from the MD simulations in this study (note that the stress, $P_{zz}$, computed by LAMMPS based on the cell volume, $V_{cell}$, has been corrected using the actual sample volume, $V_{s}$, as $P_{zz}\times V_{cell}/V_{s}$). These curves all exhibit an initial elastic regime, followed by yielding and subsequent plastic deformation, typical of MD-simulated tensile tests on MGs. The yield strengths displayed by these curves also fall within the ranges typically observed in experiments on Cu-based and Zr-based MGs. The yield strain and total strain-to-fracture appear larger than those observed in experiments on bulk MG samples but are consistent with results from some small-scale experiments on nano-sized samples. In general, MGs tend to exhibit more ductile behavior at smaller sizes, with delayed localization of strain into shear bands. Atomistic simulations, particularly MD, can be used to study the different factors (structural homogeneity, heterogeneity, sample shape, linear dimensions, aspect ratio, etc.) likely contributing to this phenomenon, which could inform and enhance the design of corresponding experiments. The main purpose of this study, however, is to investigate the fundamental compositional effects on the tensile behavior of different atomic bonds using MD simulations and statistical analysis, while keeping the sample sizes across different compositions essentially the same.

Comparing the three MG compositions in Figure 5, the yield strength decreases while the strain-to-fracture increases as the Zr content in the alloys increases from 40% to 50% and then to 60%. The post-yielding segments of the stress–strain curves exhibit crossover points that shift to higher strain with increasing Zr content in the composition. These apparent compositional effects on the overall tensile behavior of the materials stem from changes in atomic bond behavior induced by compositional variations, as will be discussed later.

**Figure 6:**
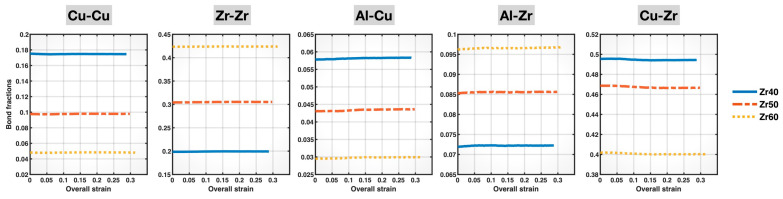
Populations (fractions) of different bonds in the Zr40, Zr50, and Zr60 MGs during the tensile test versus the overall (sample) strain.

Figure 6 displays the plots of the populations (fractions) of Cu-Cu, Zr-Zr, Al-Cu, Al-Zr, and Cu-Zr bonds in the Zr40, Zr50, and Zr60 MGs during deformation, as a function of the overall (sample) strain. All bond fractions remain virtually unchanged throughout the entire deformation process for all three alloy compositions. This means that the compositional effects on the bond fractions shown in Figure 3 remain unaffected by deformation, which simplifies our later discussion of how composition-dependent bond behavior relates to the overall mechanical behavior of the different MGs.

**Figure 7:**
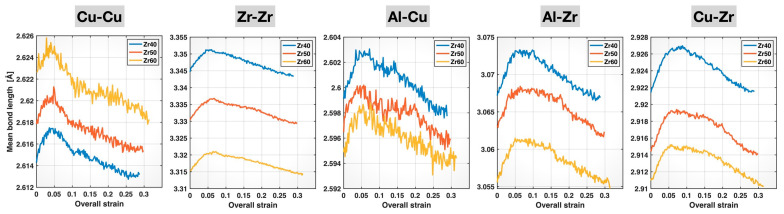
Mean bond lengths of different bonds in the Zr40, Zr50, and Zr60 MGs during the tensile test versus the overall (sample) strain.

The mean bond lengths of the Cu-Cu, Zr-Zr, Al-Cu, Al-Zr, and Cu-Zr bonds in the Zr40, Zr50, and Zr60 MGs during the deformation process are plotted in Figure 7 as a function of the overall (sample) strain. For each bond type and alloy composition, as deformation proceeds, the mean bond length initially increases nearly linearly, reaches a maximum, and then decreases continuously until fracture. These main characteristics are consistent with our previous findings in the Cu_46_Zr_47_Al_7_ MG [21]. In terms of compositional effects, Figure 7 shows that, for a fixed bond type, the comparison of the mean bond lengths across the Zr40, Zr50, and Zr60 MGs remains unchanged during deformation. The Zr60 MG consistently shows the longest mean bond length for Cu-Cu and the shortest mean bond lengths for Zr-Zr, Al-Cu, Al-Zr, and Cu-Zr among the three alloy compositions throughout the deformation process. In other words, the bond shortening of Zr-Zr, Al-Cu, Al-Zr, and Cu-Zr, and the bond elongation of Cu-Cu, caused by increasing Zr content in the alloy composition, as shown earlier in Figure 4, persist during deformation.

**Figure 8:**
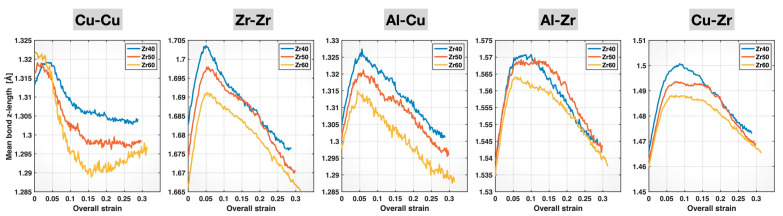
Mean bond z-lengths of different bonds in the Zr40, Zr50, and Zr60 MGs during the tensile test versus the overall (sample) strain.

The mean bond length data are not the most suitable for revealing changes in the tensile behavior of the various bonds, as they are not calculated specifically along the loading (z-) direction. Thus, we computed the mean bond z-lengths (lengths projected onto the z-direction) for the different bond types and alloy compositions, and we plot them in Figure 8 as a function of the overall (sample) strain. Several important trends can be observed in Figure 8, as the composition varies from Zr40 to Zr50 and then to Zr60. First, the initial elastic stretching portion of these plots gradually diminishes for Cu-Cu, Al-Cu, Al-Zr, and Cu-Zr bonds and completely disappears for Cu-Cu in Zr60. Second, bond yielding (signified by the cessation of the initial bond stretching) occurs earlier (i.e., at lower sample strains) for these same four bond types, particularly for Cu-Cu bonds in Zr60, which yield almost immediately after the start of the tensile test. Both of these observations indicate that the Cu-Cu, Al-Cu, Al-Zr, and Cu-Zr bonds all become weaker and less elastic with increasing Zr content, at least up until bond yielding. For the Zr-Zr bonds, a subtle shift in bond yielding toward higher sample strains with increasing Zr content is observed. Meanwhile, the elastic stretching segment of the Zr-Zr mean bond z-length curves appears to extend further with rising Zr content. These characteristics indicate that the Zr-Zr bonds become stronger and more elastic, at least up until bond yielding, as the alloy composition varies from Zr40 to Zr60.

**Figure 9:**
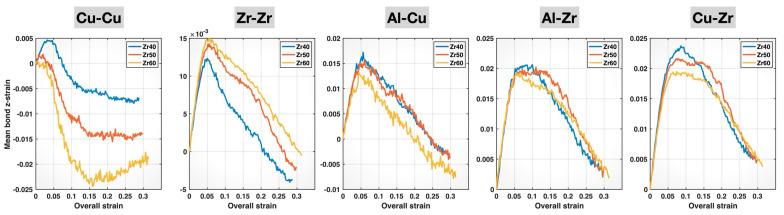
Mean bond z-strains of different bonds in the Zr40, Zr50, and Zr60 MGs during the tensile test versus the overall (sample) strain.

To account for differences in the initial mean bond z-lengths for each bond type across the different MGs, we further calculated the mean bond z-strain (i.e., the relative change in the mean bond z-length during deformation, with respect to its initial value) for each bond type and alloy composition. The results are plotted in Figure 9. The compositional effects on the bond behavior are now more evident, as the initial elastic stretching portions of the curves for the three compositions align for each bond type. These mean bond z-strain curves further confirm the composition-induced weakening of the Cu-Cu, Al-Cu, Al-Zr, and Cu-Zr bonds and strengthening of the Zr-Zr bonds up until bond yielding. The weakening of Cu-Cu bonds and the strengthening of Zr-Zr bonds are consistent with their respective bond elongation and bond shortening induced by higher Zr content in the composition, as observed in Figure 4 and Figure 7. These effects indeed continue to dominate the behavior of these two bond types even after bond yielding, until sample fracture. For Cu-Cu bonds, the Zr60 alloy exhibits the most negative post-yielding mean bond z-strain among the three compositions, indicating the greatest plastic characteristic (dominated by bond reconstruction) or, in other words, the weakest elastic characteristic. For Zr-Zr bonds, the mean bond z-strain curve of the Zr60 alloy remains the highest among the three compositions up to sample fracture, suggesting the strongest elastic characteristic.

Compared to Cu-Cu and Zr-Zr bonds, the compositional effects on Al-Cu, Al-Zr, and Cu-Zr bonds are more complex. Up until bond yielding, these bonds exhibit weakened elastic behavior with increasing Zr content in the alloy composition, stretching to and yielding at lower bond z-strains (as well as lower sample strains). This is surprising because these bonds are shortened with more Zr, as shown in Figure 4 and Figure 7. The weakened elastic behavior of Al-Cu bonds with increasing Zr content persists beyond bond yielding and through to sample fracture, as evidenced by the consistently lowest-lying Al-Cu mean bond z-strain curve for the Zr60 alloy. In contrast, the post-yielding mean bond z-strain curves of Al-Zr and Cu-Zr bonds display crossover points among the Zr40, Zr50, and Zr60 alloys, indicating the longer retention of the bond strength at higher Zr content—likely related to the composition-induced bond shortening in these bonds. The lack of crossover points among the post-yielding Al-Cu mean bond z-strain curves for the three MGs may be attributed to the mildest bond-shortening effect of composition on these bonds, as evidenced by the least negative slope of the Al-Cu curve in Figure 4.

**Figure 10:**
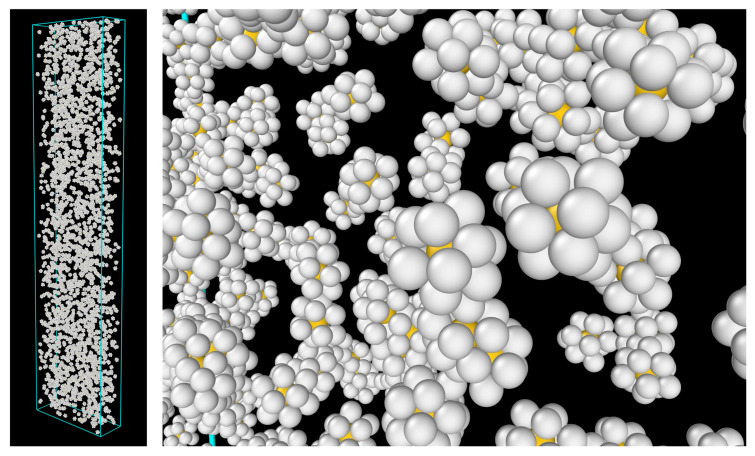
(Perspective view) ICO clusters (core and shell) in the Zr60 MG before the tensile test. (**Left**): overall view; (**Right**): close-up view. Color code: yellow—core atoms; gray—shell atoms.

To understand the surprising weakening of the Al-Cu, Al-Zr, and Cu-Zr bonds during the elastic stretching stage with increasing Zr content, we examined the short-range ordering in the atomic configurations of the three MGs—more specifically, the ICO clusters, representative images of which are shown in Figure 10. These clusters are known to exist in many MGs, serving as a structural mechanism that helps to stabilize the overall disordered material (including both the solid and supercooled liquid states) [24]. They exhibit distinctive characteristics, such as five-fold rotational symmetry and an inverted core–shell potential energy landscape—that is, lower energy on the shell than at the core [25]. They are also known to be more resistant to deformation than the surrounding disordered glassy matrix [26,27,28,29].

**Figure 11:**
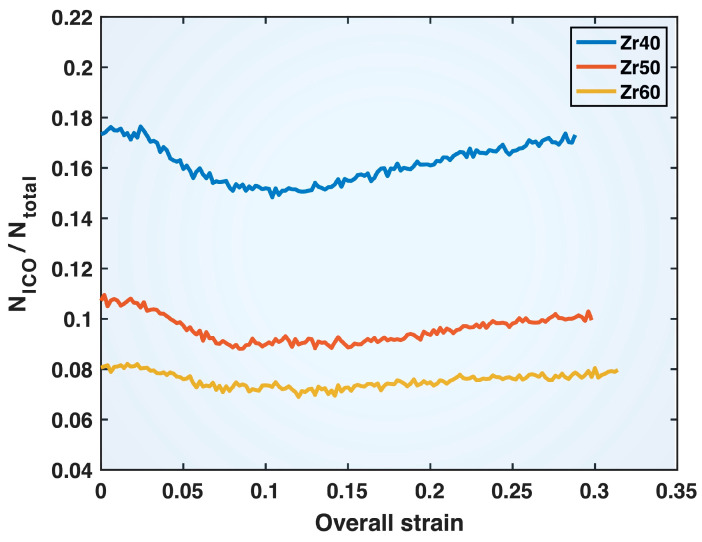
Fraction of ICO cluster atoms (both cores and shells), N_ICO_, among the total number of atoms, N_total_, in the Zr40, Zr50, and Zr60 MGs plotted versus the overall (sample) strain.

Figure 11 shows the fraction of ICO cluster atoms (both cores and shells), N_ICO_, among the total number of atoms, N_total_, in the Zr40, Zr50, and Zr60 MGs during deformation as a function of the overall (sample) strain. It is evident that the ICO atomic fraction decreases with increasing Zr content in the alloy composition, a trend that can be expected to contribute to the overall weakening of the material. Additionally, it can be seen from Figure 11 that, in the elastic regime of deformation (up to ~0.07 sample strain) of all three MGs, the ICO atomic fraction drops noticeably. This alludes to the role of ICO clusters in resisting deformation and contributing to the strength of MGs.

**Figure 12:**
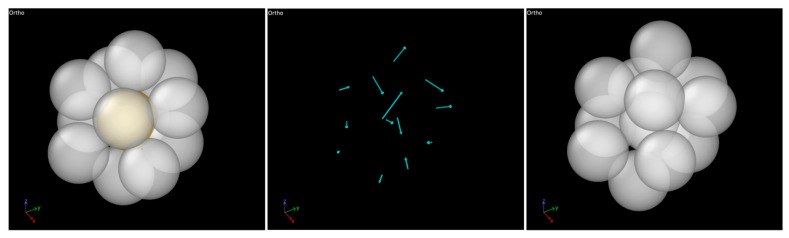
MD snapshots showing the transformation of an ICO cluster (**left**) into a non-ICO cluster (**right**) due to deformation (over a 0.2% sample strain increment), with the displacement vectors of the atoms shown in the (**middle**).

Figure 12 provides representative snapshots illustrating how an ICO cluster transforms into a non-ICO cluster due to deformation. As shown in the middle panel of Figure 12, the displacement vectors of the atoms, determined by comparing the two configurations of the cluster, exhibit highly non-uniform directions and magnitudes, indicating a non-affine transformation of the atomic positions in response to the external stress. This non-affine transformation leads to the loss of ICO characteristics as the cluster evolves into the new configuration. One may expect the ICO clusters in the different alloy compositions to possess varying stability against mechanical deformation. The mechanical stability of ICO clusters can be quantified by the relative (percent) drop in the ICO cluster fraction from the stress-free state to the minimum value observed during deformation. It is calculated to be 15%, 18%, and 14% for Zr40, Zr50, and Zr60, respectively. Both these values and the unchanged relative positions of the three curves in Figure 11 throughout the deformation process indicate that variations in the mechanical stability of ICO clusters are a minor effect of composition changes, compared to the more significant variation in the ICO cluster fraction (which decreases with increasing Zr content).

**Figure 13:**
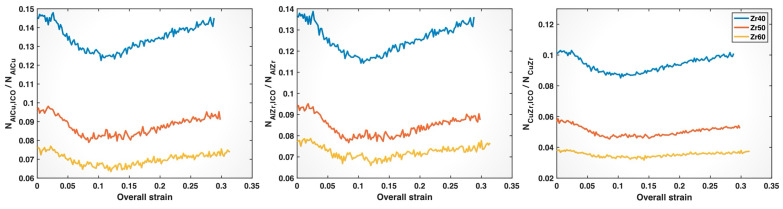
Fractions of Al-Cu, Al-Zr, and Cu-Zr bonds associated with icosahedral clusters among the respective total numbers of bonds in the Zr40, Zr50, and Zr60 MGs plotted versus the overall (sample) strain.

To more directly relate the impact of the composition on ICO clusters to the weakening of Al-Cu, Al-Zr, and Cu-Zr bonds with increasing Zr content, we determined the fractions of these bonds associated with ICO clusters (i.e., formed by ICO atoms) among their respective total numbers of bonds in the three MGs. It is interesting to note that the ICO clusters in all three MGs have cores that are mostly (>90%) occupied by Cu or Al, while the shell atoms include all three elements, Cu, Zr, and Al, with proportions more closely matching the nominal alloy compositions. As shown in Figure 13, with increasing Zr content, the ICO-associated fractions of Al-Cu, Al-Zr, and Cu-Zr bonds all decline, or, in other words, a larger proportion of these bonds exist in the softer, more disordered glassy matrix. This provides a plausible explanation for the composition-induced pre-yielding weakening of these bonds observed in Figure 8 and Figure 9, despite the bond shortening shown in Figure 4 and Figure 7. After bond yielding, bond shortening may play an increasingly important role, particularly for Al-Zr and Cu-Zr bonds, although the reduced association with ICO clusters may still contribute. As discussed earlier, the composition-induced bond shortening is likely responsible for the crossover observed in the post-yielding mean bond z-strain curves for Al-Zr and Cu-Zr bonds, despite their reduced association with ICO clusters in Zr50 and Zr60. In the case of Al-Cu bonds, the composition-induced bond shortening is the mildest among all bond types, as shown in Figure 4. As a result, the post-yielding mean bond z-strain curves for Al-Cu bonds in Zr50 and Zr60 remain dominated by the reduced association with ICO clusters and, hence, do not exhibit a crossover with the curve for Zr40.

It is noted that the information presented in Figure 10, Figure 11, Figure 12 and Figure 13 was obtained from the ICO clusters, each consisting of one core atom surrounded by twelve shell atoms, as identified using the polyhedral template matching method in the OVITO program (see Section 3 for more details). Some of these ICO clusters are isolated, while others are connected through sharing one or more atoms. The above results include all identified ICO clusters, both isolated and connected.

The compositional effects on the various atomic bonds can now be used to understand the differences in the overall deformation behavior among the three MGs shown in Figure 5. As reported in our previous study [21], the elastic deformation and yielding conditions (i.e., yield strength and strain) of an MG are mainly determined by the intermediate bonds (Zr-Zr and Al-Cu) and the strong bonds (Al-Zr and Cu-Zr), while the post-yielding residual strength and continued deformability are predominantly governed by the strong bonds. Thus, the weakened elastic and yielding behavior of the alloys with increasing Zr content in Figure 5 can be viewed as the net result of strengthened Zr-Zr bonds and weakened Al-Cu, Al-Zr, and Cu-Zr bonds. The effect of Zr-Zr bonds is counteracted by those of the other three bond types, likely due to two reasons. First, Zr-Zr bonds constitute ~20%, 30%, and 42% of all bonds in Zr40, Zr50, and Zr60, respectively—less than the combined fraction of the other three types (~63% in Zr40, 60% in Zr50, and 53% in Zr60). Second, Zr-Zr bonds are an intermediate-strength bond type, weaker than Al-Zr and Cu-Zr bonds.

The post-yielding crossover of the stress–strain curves of the three MGs in Figure 5, along with the shift in the crossover points to later stages of deformation with increasing Zr content, resembles the bond behavior shown in Figure 9 for the strong bond types, Al-Zr and Cu-Zr. Thus, these features can be interpreted as a manifestation of the compositional effects on the behavior of the strong bonds—after bond yielding.

## 3. Methodology

The overall methodology in this study is similar to that in our recent publication [21]: large-scale MD simulations were used to create MG samples with the target compositions and to conduct uniaxial tensile testing, while visualization and statistical analysis were performed to characterize the atomic structure and atomic bonds based on the atomic- and system-level data exported from the MD simulations.

For the MD simulations, the widely adopted open-source software LAMMPS (Large-Scale Atomic/Molecular Massively Parallel Simulator, ver. 3/3/2020) [30,31] was used in conjunction with a ternary Embedded Atom Method (EAM) interatomic potential specifically developed for the Cu-Zr-Al system [32]. A bulk Cu FCC crystal comprising 864,000 atoms (30 by 40 by 180 lattice units) was first melted at 1600 K under the NPT (controlled number of particles, N, pressure, P, and temperature, T) ensemble, with all (x-, y-, and z-) periodic boundary conditions. Subsequently, some of the atoms in the liquid Cu were randomly replaced with appropriate amounts of Zr and Al to create the target alloy compositions used in this study: Cu_53_Zr_40_Al_7_ (Zr40), Cu_43_Zr_50_Al_7_ (Zr50), and Cu_33_Zr_60_Al_7_ (Zr60). Each alloy sample was relaxed at 1600 K for 50 ps to reach the equilibrium molten state and then cooled down to 300 K at a rate of 1 K/ps to obtain the corresponding MG (with dimensions of 12.2–12.7, 16.2–16.9, and 73.0–76.3 nm in the x-, y-, and z-directions, respectively).

Next, the x-dimension of each MG sample was trimmed down to 5.5 nm by deleting atoms so as to better approximate the overall sample aspect ratios commonly used in tensile experiments. With the periodic boundary conditions turned off in the x- and y-directions, each sample was relaxed at 300 K for 50 ps and then subjected to uniaxial tensile loading at a constant strain rate of 10^−4^ /ps along the z-direction until fracture. The Nose–Hoover thermostat was used to maintain the sample temperature at ~300 K, and atomic coordinates along with system data (e.g., cell volume, overall stress ($P_{zz}$) on the sample) were periodically exported during the tensile test.

After the MD simulations were completed, the OVITO (Open VIsualization TOol, ver. 2.9.0) software [33] was used to visualize the atomic structure and perform certain data analyses (e.g., calculating overall and partial radial distribution functions (RDFs), finding actual sample volume, identifying core and shell atoms of ICO clusters, and counting various types of atomic bonds among ICO atoms). The actual sample volume was determined using the Construct Surface Mesh modifier. ICO core atoms were identified using the polyhedral template matching method, and their twelve nearest neighbors were subsequently identified as the ICO shell atoms.

MATLAB (version: R2023a) and custom scripts were utilized to perform a statistical analysis of the bond populations, bond lengths, bond z-lengths, and bond z-strains, where the bonding atoms were identified based on the cutoff distances taken from the valley between the first and second peaks on the partial RDF curves for the target atomic pairs (e.g., Cu-Cu, Zr-Cu). The cutoff distance for any bond type in this study was virtually invariant across the three MG compositions (as shown in the Section 2) and did not show appreciable changes during the tensile testing (similar to what was observed in our previous study [21]). For each bond type within each deformation state of each sample, the mean bond length and mean bond z-length were calculated by averaging the corresponding values over all identified bonds of that type. The mean bond z-strain was then computed as the relative change in the mean bond z-length with respect to the initial (undeformed) state before tensile loading.

## 4. Conclusions

We have used our recently developed computational method to investigate the compositional effects on the tensile behavior of various atomic bonds in multicomponent Cu_93−x_Zr_x_Al_7_ (x = 40, 50, 60 at.%) MGs. The focus has been placed on the bond fractions, mean bond lengths, mean bond z-lengths, and mean bond z-strains. We have also examined ICO short-range ordering in the MGs in relation to the composition-dependent bond behavior and discussed the connection between compositional effects on bond behavior and variations in the overall deformation characteristics of the different MGs. The key findings from this study are summarized as follows:

(1) Increasing Zr content in the composition (while decreasing Cu and fixing Al) leads to higher fractions of Zr-Zr and Al-Zr bonds and lower fractions of Cu-Cu, Al-Cu, and Cu-Zr bonds. The bond fractions in each MG remain nearly constant throughout the deformation process. The Al-Al bonds in all three MGs constitute a very small fraction (~0.1%) and are therefore not discussed in this study.
(2) Increasing Zr content in the composition causes the shortening of Zr-Zr, Al-Zr, Cu-Zr, and Al-Cu bonds and elongation of Cu-Cu bonds, as indicated by the mean bond lengths. These effects persist throughout the deformation process.
(3) The mean bond z-length and z-strain data for the higher-Zr-content compositions show the earlier yielding (i.e., weakened elastic behavior) of Cu-Cu bonds and delayed yielding (i.e., strengthened elastic behavior) of Zr-Zr bonds, consistent with the elongation of Cu-Cu bonds and the shortening of Zr-Zr bonds revealed by their mean bond length data.
(4) The mean bond z-length and z-strain data show the earlier yielding (i.e., weakened elastic behavior) of Al-Cu, Al-Zr, and Cu-Zr bonds in the higher-Zr-content compositions, despite the bond shortening revealed by their mean bond length data. This apparent paradox can be reconciled by the reduced association of the bonds with ICO clusters as the Zr content increases.
(5) The post-yielding mean bond z-strain curves of the Al-Zr and Cu-Zr bonds exhibit crossover points among the different compositions, which shift to later stages of deformation (i.e., higher sample strain) with increasing Zr content.
(6) The overall stress–strain curves of the three MGs display weaker elastic behavior and easier yielding at higher Zr content, which can be related to the compositional effects on the elastic and yielding behavior of Al-Cu, Al-Zr, and Cu-Zr bonds.
(7) The post-yielding stress–strain curves of the three MGs display crossover points that shift to later stages of deformation with increasing Zr content. This can be attributed to the compositional effects on the post-yielding behavior of the Al-Zr and Cu-Zr bonds.

Additionally, our results reveal that the mean bond length of any bond type does not coincide with the first peak position of the corresponding partial RDF curve. Instead, it is better represented by the g(r)-weighted average of r over the range of the first peak, computed via integration or approximating summation.

## Data Availability

The original contributions presented in this study are included in the article. Further inquiries can be directed to the corresponding author.
